# Multidimensional predictors of physical frailty in older people: identifying how and for whom they exert their effects

**DOI:** 10.1007/s10522-017-9677-9

**Published:** 2017-02-03

**Authors:** Yew Y. Ding, Jouni Kuha, Michael Murphy

**Affiliations:** 1 Department of Methodology, London School of Economics, Columbia House, Houghton Street, London, WC2A 2AE UK; 2 Department of Statistics, London School of Economics, London, UK; 3 Department of Social Policy, London School of Economics, London, UK; 4 Department of Geriatric Medicine & Institute of Geriatrics and Active Ageing, Tan Tock Seng Hospital, 11 Jalan Tan Tock Seng, Singapore, 308433 Singapore

**Keywords:** Aged, Mediators, Moderators, Growth curve, Allostatic load, Social support, Social integration

## Abstract

Physical frailty in older people is an escalating health and social challenge. We investigate its physical, psychological, and social predictors, including *how* and *for whom* these conditions exert their effects. For 4638 respondents aged 65–89 years from wave 2 of the English Longitudinal Study of Ageing, we examine prediction of future physical frailty by physical, psychological, and social conditions using latent growth curve analysis with multiple indicators. In addition, we explore their indirect effects through disease and physiologic decline, and repeat these analyses after stratification by gender, age group, and selected conditions which are possible moderators. We find that chronic disease, allostatic load, low physical activity, depressive symptoms, cognitive impairment, and poor social support all predict future physical frailty. Furthermore, chronic disease and allostatic load mediate the effects of low physical activity, depressive symptoms, and cognitive impairment on future physical frailty. Finally, although poor social integration is not a predictor of future physical frailty, this condition moderates the indirect effect of poor social support through chronic disease by rendering it stronger. By virtue of their roles as predictor, mediator, or moderator on pathways to physical frailty, chronic disease, allostatic load, low physical activity, cognitive impairment, depressive symptoms, poor social support, and poor social integration are potentially modifiable target conditions for population-level health and social interventions to reduce future physical frailty in older people.

**Electronic supplementary material:**

The online version of this article (doi:10.1007/s10522-017-9677-9) contains supplementary material, which is available to authorized users.

## Introduction

### Background

Frailty denotes the multidimensional loss of an individual’s reserves that occurs with greater probability with advancing age, and results in vulnerability to developing adverse outcomes (Lally and Crome 2007). In biomedical circles, frailty is widely considered to be a clinical syndrome with an underlying biological basis, and is thought to be a transitional state between robustness and functional decline (Lang et al. 2009). Its prevalence from different studies that used a range of frailty instruments yielded an estimate of 10.7% among adults aged 65 years and older (Collard et al. 2012). Thus, one out of every 10 community-dwelling older people is frail. Frailty confers increased risk of adverse health outcomes that matter to older people which include death (Buchman et al. 2009; Cawthon et al. 2007; Gu et al. 2009; Mitnitski et al. 2004; Rockwood et al. 2011), disability (Avila-Funes et al. 2008; Romero-Ortuno et al. 2011; Woo et al. 2006), falls (Bilotta et al. 2012; Samper-Ternent et al. 2012), cognitive impairment and dementia (Auyeung et al. 2011; Boyle et al. 2010; Woo et al. 2006), lower health-related quality of life (Kanauchi et al. 2008), hospitalization (Bilotta et al. 2012), greater health services utilization (Rockwood et al. 2011), and institutionalization in long-term care facilities (Jones et al. 2005). In view of these consequences, frailty plays a central role in the well-being of older people at the individual and societal levels, and has major public health importance. Moreover, with the projection of rapid growth in number of older people living across the world, frailty presents a rapidly escalating societal challenge on a global scale (Conroy 2009). Given its impact, frailty has been described as the most problematic expression of ageing (Clegg et al. 2013).

On a more positive note, accumulating evidence suggests that frailty is addressable. For example, targeted interventions such as exercise have shown promise in reducing incident frailty in selected groups of older people (Mohandas et al. 2011). Indeed, reducing frailty at the population level is a desirable goal. To this end, a more precise understanding of predictors of frailty holds the key to delaying its onset and slowing its progression. This knowledge can in turn assist in informing the formulation of health and social policies which address frailty in older people.

### Physical predictors

Research on frailty over the past two decades has yielded important information on its predictors. To date, most of the available evidence concerns the physical domain. For example, older age (Fallah et al. 2011; Ottenbacher et al. 2009) and female gender increase the likelihood of developing frailty (Etman et al. 2012; Peek et al. 2012; Woods et al. 2005). Genetic factors play an important role with data from multi-generational families suggesting that its contribution is comparable with that of environmental factors (Garibotti et al. 2006). Chronic disease (Ottenbacher et al. 2009; Strawbridge et al. 1998; Syddall et al. 2010; Woods et al. 2005), allostatic load (Gruenewald et al. 2009), and chronic systemic inflammation (Barzilay et al. 2007) are medical conditions associated with developing frailty. Low physical activity (Strawbridge et al. 1998), being either underweight, overweight, or obese (Woods et al. 2005), smoking (Woods et al. 2005) and heavy drinking (Strawbridge et al. 1998) are lifestyle-related conditions that also increase the risk of frailty.

### Psychological and social predictors

Beyond the physical domain, lower cognition and depression are psychological conditions that confer higher risk of incident frailty (Ottenbacher et al. 2009; Strawbridge et al. 1998; Woods et al. 2005). In the social realm, having less education and lower income, non-white collar occupation, living alone, and being social isolated are all associated with increased risk of developing frailty or worsening of frailty (Alvarado et al. 2008; Etman et al. 2012; Peek et al. 2012; Strawbridge et al. 1998; Syddall et al. 2010; Woods et al. 2005). Financial strain also increases this risk (Alvarado et al. 2008; Peek et al. 2012). These conditions reflect chronic stressors. From a life course perspective, poor social conditions in childhood such as experiencing hunger and having challenging socioeconomic circumstances also increases the risk of developing frailty (Alvarado et al. 2008). Conversely, social support characterized by perceived emotional support from family or friends protects against increasing degrees of frailty (Peek et al. 2012). Participation in group activities also confers lower risk of incident frailty in older persons (Fushiki et al. 2012).

### Pathways to frailty

More recently, a life course approach was proposed to offer a more comprehensive framework for investigating determinants and effects of frailty in older people. It attempts to integrate rather than segregate biological and social risk factors (Kuh 2007). Typically, there is explicit temporal ordering of exposures and inter-relationships among these variables. Their effects are either direct or through intermediate conditions, also designated as mediators. A tangible output is a set of pathways for these conditions which serves as a suitable framework for the application of statistical modeling techniques such as structural equation modeling (Ben-Shlomo and Kuh 2002).

**Fig. 1:**
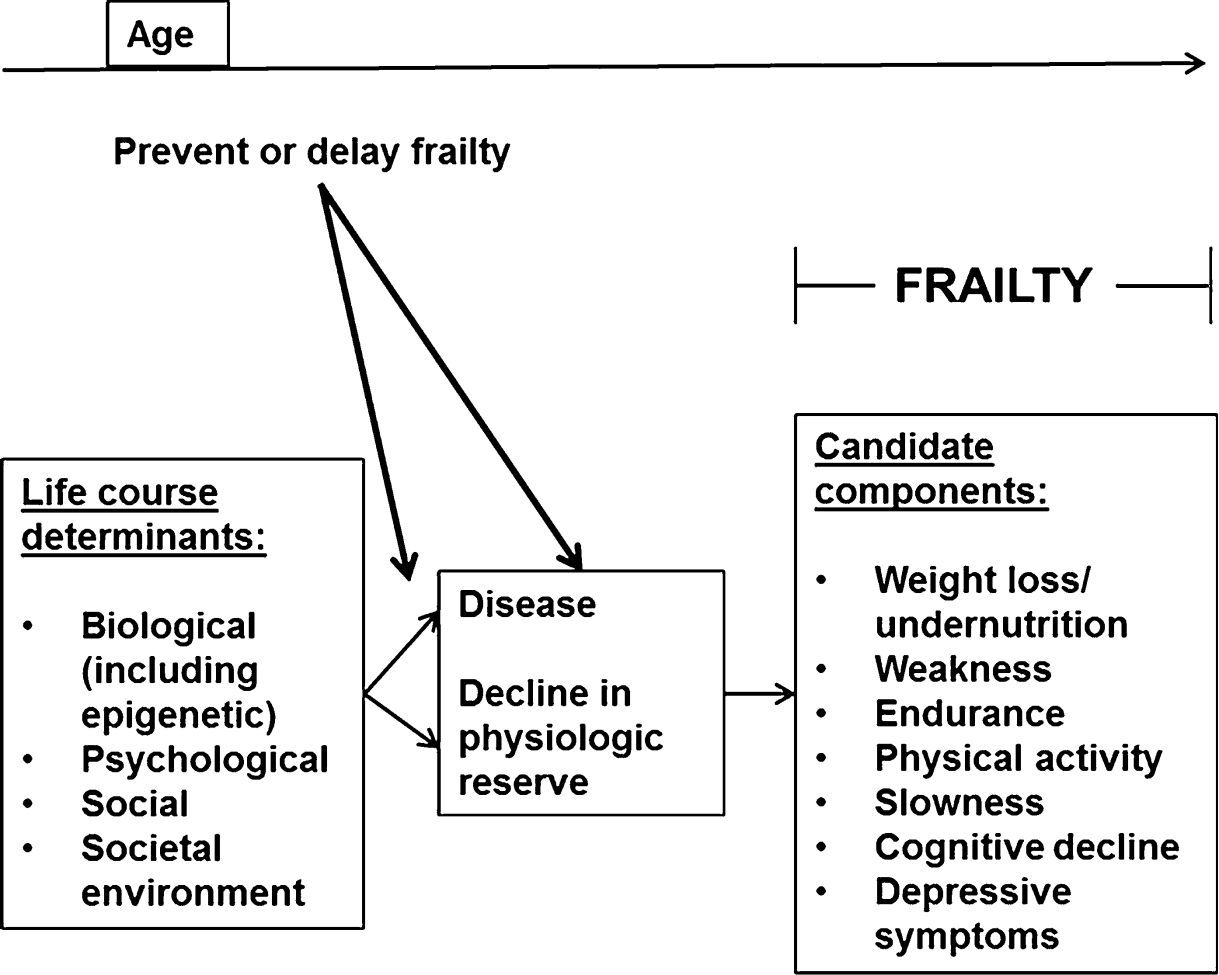
Working framework of the Canadian Initiative for Frailty and Aging (adapted from Bergman et al. (2004) with modifications)

Adopting a life course approach, Bergman developed the working framework of the Canadian Initiative for Frailty and Aging which provides a graphical representation of multidimensional exposures across the life span (Bergman et al. 2004). An adapted version of this framework showing pathways to frailty and including its physical, psychological, and social determinants is shown in Fig. 1. Their effects are mediated by disease and physiologic reserve decline. This framework offers a useful starting point for assembling a set of predictors on pathways to physical frailty in older people. To date however, empirical studies examining this framework have not yet been reported.

Building on the Canadian framework, the integral conceptual model of frailty was subsequently proposed (Gobbens et al. 2010). Here, frailty is explicitly specified as having distinct physical, psychological, and social domains. This allows physical frailty to be disaggregated from the other two frailty domains, thereby permitting less constrained exploration of the relationship of frailty with its multidimensional predictors. Adopting this approach to specifying frailty, a physical frailty specification with three indicators, namely, slowness, weakness and exhaustion was developed and its construct and concurrent validity demonstrated (Ding 2016).

### Research questions

Following this review, we study pathways to frailty as hypothesized in the working framework of the Canadian Initiative for Frailty and Aging with three research questions in mind. Our first question focuses on key multidimensional conditions that predict physical frailty. More specifically, what are the effect sizes of physical, psychological, and social predictors of physical frailty controlling for the effects of each other? Our second question concerns *for whom* these multidimensional predictors exert their effects. In particular, to what extent are the effects of predictors influenced by other predictors? Our third question examines *how* these predictors exert their effects. More precisely, are the effects of predictors mediated by disease and decline in physiological reserve as suggested by the working framework of the Canadian Initiative for Frailty and Aging? In answering these questions, we seek to advance beyond merely confirming that specific physical, psychological, and social conditions are predictors of physical frailty, to further estimating their effects over and above each other. In addition, we examine the roles of key conditions in moderating the effects of other conditions and in mediating indirect effects. To this end, we will operationalize the aforementioned physical frailty specification with three indicators and use it in the analysis of panel data of older people from the English Longitudinal Study of Ageing (ELSA). ELSA is an ongoing longitudinal survey of a representative sample of the English population aged 50 years and older living in their homes at baseline (Steptoe et al. 2013). It offers a broad range of reliable and multidimensional data across biennial waves beginning from 2002.

## Methods

### Study population

Our study population comprises 4638 respondents aged 65–89 years at wave 2 (2004) of ELSA (Marmot et al. 2015). Those aged 90 years and older are excluded because their age is uniformly coded as “90”. All respondents gave informed consent. Ethical approval for ELSA was granted by the Multicenter Research and Ethics Committee. Ethical oversight for this study is provided by procedures of the London School of Economics Ethics Policy.

### Frailty measures

Physical frailty is specified by three indicators drawn from those of the Cardiovascular Health Study (CHS) frailty phenotype (Fried et al. 2001), namely slowness, weakness, and exhaustion at waves 2 (2004), 4 (2008), and 6 (2012). *Slowness* is operationalized as the average gait speed (in m/s) of two attempts at walking a distance of 2.4 m, but with values reversed through multiplication by −1. *Weakness* is measured by the dominant hand grip strength in kg, which is multiplied by 1.5 for women. The differential handling of raw grip strength values in men and women is based on gender-specific and population-independent values for grip strength proposed for the CHS frailty phenotype criteria (Saum et al. 2012). After that, values are reversed through multiplying them by −1. *Exhaustion* is a binary variable based on a positive response to at least one of two items in the Center for Epidemiologic Studies Depression Scale (CES-D scale) on whether the respondent “felt everything they did during the past week was an effort” and “could not get going much of the time in the past week” (Radloff 1977). From among different permutations of the five components of the CHS frailty phenotype, the combination of these three indicators has been shown and argued to be preferred in representing the physical frailty construct for investigation of frailty pathways (Ding 2016). Confirmatory factor analysis (CFA) with these three indicators for waves 2, 4, and 6 is performed while assuming and therefore, imposing scalar (strong) invariance over time where all three loadings and intercepts are held constant across time. This measurement model is then incorporated in the full structural model. In addition, unique physical frailty factor scores for each respondent are derived at the three time points and then utilized to describe the study population.

To further describe frailty status in our study population, a 30-item frailty index (FI) based on a deficit accumulation approach is constructed (scoring system in Supplementary Materials) and represented as a scalar measure ranging from 0 to 1 (Mitnitski et al. 2001). Using cut-off values in accordance with previous reports, FI values of at least 0.25 define frailty (Rockwood et al. 2007).

### Variables

Physical frailty is the outcome of interest that is specified at waves 2, 4 and 6 as factors with multiple indicators on a latent growth curve. Based on the Canadian working framework and evidence assembled from the literature, physical, psychological, and social conditions are shortlisted for inclusion as predictors in our models. Beyond *age* and *gender*, physical predictors include *obesity* (binary: body mass index (BMI) of 30 kg/m^2^ or more with reference to BMI less than 30 kg/m^2^ but more than 20 kg/m^2^), being *underweight* (binary: BMI of 20 kg/m^2^ or less with reference to BMI less than 30 kg/m^2^ but more than 20 kg/m^2^), *low physical activity* (four levels of decreasing intensity activity related to occupation and exercise), *chronic disease* (count of conditions from 0 to 14), *allostatic load* (score of 0–9), *smoking* history (binary: whether ever smoked), and *high alcohol intake* (binary: whether had alcohol drink almost every day in the past 12 months). Allostatic load reflects physiological dysregulation in multiple body systems and is specified by nine biomarkers including blood pressure readings, anthropometric measurements, and blood tests for cholesterol levels, glucose control, and inflammatory markers (Gruenewald et al. 2009). For each biomarker, a score of one is awarded for values beyond a cut-off level reflecting high risk, with a score of zero given if otherwise. Scoring systems for chronic disease and allostatic load are provided in Supplementary Materials.

Psychological predictors include *depressive symptoms* which are based on a count of six out of eight items (score of 0–6) of the CESD Scale. The two omitted items are those already used to specify exhaustion as a physical frailty indicator. *Cognitive impairment* is measured by reversing a cognitive index based on the combined memory and executive function test performance (score of 0–49).

Social predictors include *low education* (binary: no qualifications compared with any qualification), and *low wealth* (binary: lowest 2 deciles compared with highest 8 deciles of non-pension wealth). Additionally, *poor social integration* reflecting social isolation is based on a combined score on five items (score of 0–14) concerning whether respondents have no spouse or partner living with them, had little contact with children, had little contact with other family members, had little contact with friends, and were not a member of any organization, club or society. Contact includes meeting, phoning, and writing or email. Its precise specification is adapted from that of a previous study (Banks et al. 2010). Finally, *poor social support*, in terms of deficient emotional support, and reflecting negative social interaction with family and friends is measured by the combined scores on three items each on lack of positive support, and occurrence of negative support (score of 0–54). Lack of positive support is measured by disagreement with statements on “understand the way you feel”, “can rely on if you had a serious problem”, and “can open up to them if you need to talk” with respect to children, other family members, and friends. Negative support is measured by agreement with statements on whether children, other family members, and friends “criticizes the respondent”, “lets the respondent down”, and “gets on the nerves of respondent”. This specification is again based on the aforementioned previous study (Banks et al. 2010). Scoring systems for poor social integration and poor social support are provided in Supplementary Materials. Social vulnerability, which is a broader description of an individual’s social circumstances (Andrew et al. 2008) is not included given that it arguably encompasses multiple key social constructs.

### Statistical analyses

A series of structural equation models using latent growth curve analysis (Newsom 2015) are developed to examine the effect of predictors on physical frailty. The growth curve is specified as linear and measured by multiple indicators for physical frailty at waves 2, 4, and 6. Random effects capture inter-individual differences in physical frailty development that are conceptualized as two growth factors. The first is the intercept growth factor which reflects physical frailty at wave 2 and represents inter-individual differences in initial physical frailty at wave 2. The other is the slope growth factor which reflects physical frailty change across waves 2–6, and represents inter-individual differences in physical frailty trajectory over time.

Model 1 concerns prediction of initial physical frailty and its change over time. It comprises two parts. The first part is the regression of intercept and slope factors for physical frailty on predictors designated as time-invariant variables, such as age (at wave 2) and gender. Other predictors not expected to change over the three time points for the vast majority of respondents are smoking history, high alcohol intake, low education level, and low wealth. Obesity is also designated as time-invariant, given that BMI data are not always available at the three time points. The second part is the regression of physical frailty factors at waves 2, 4, and 6 on their lagged time-varying predictors, namely chronic disease, allostatic load, low physical activity, depressive symptoms, cognitive impairment, poor social support, and poor social integration measured at waves 1, 2, and 4 respectively. Wave 1 is used given that data is not available for six out of seven of these variables at wave 0. In addition, stratified analyses according to gender and age group (below 75 years and at least 75 years) are performed. Model 2 extends Model 1 by examining moderation of the effects of predictors on physical frailty by low physical activity, depressive symptoms, poor social support, and poor social integration using stratified analyses of two subgroups defined by whether values are below or above their mean values. Equivalent effects across time are constrained to be equal.

Model 3 extends Model 1 by including mediation of the effects of predictors on change in physical frailty. The indirect effects of time-varying predictors at waves 1, 2, and 4 on physical frailty factor at waves 2, 4, and 6 that are mediated by chronic disease and allostatic load at waves 2, 4, and 6 are of interest. These indirect effects are estimated by obtaining the product of the coefficients of the predictor-mediator and mediator-outcome effects, and then using Sobel’s test to test their significance (Sobel 1982). Gender and age group-specific effects are also estimated with stratified analyses. Absence of predictor-mediator interaction is assumed. Finally, Model 4 extends Model 3 by including stratified analyses to explore moderation of these indirect effects (moderated mediation) by the four conditions examined in Model 2,

Mathematical equations for Models 1–4, as well as graphical representations of Models 1 and 3 are provided in Supplementary Materials (Figs. 3 and 4 respectively for the latter). The models are estimated using maximum likelihood with robust standard errors (MLR). Missing values for dependent variables due to both attrition and item non-response are handled by full information maximum likelihood (FIML) with the assumption of missing at random (MAR). FIML is a procedure that is analogous to multiple imputation but without actual creation of imputation datasets. Rather, missing data is handled within the analysis model using maximum likelihood estimation which identifies population parameters having the highest probability of producing the sample data. It uses all available data to generate estimates and assumes multivariate normality. It is also implemented for predictor variables by treating them as dependent variables through estimating their sample means.

Sensitivity analysis is explored in two ways. Firstly, the MAR assumption is relaxed to consider the possibility that missing values for the outcome variable are missing not at random (MNAR). This is particularly relevant given that missing values due to death or drop out may be MNAR. To perform this, Wu and Carroll’s selection model (Enders 2011) which is a shared parameter model that is conditional on the latent factors, is incorporated to explore the extent to which results change when MNAR is considered. Graphical representation of Model 1 incorporating this selection model is shown in Fig. 5 of Supplementary Materials. Secondly, depressive symptoms are measured by the full set of eight items of the CESD instrument rather than just the six selected items.

Mplus version 7.4 (Muthén et al. 1998–2012) is used to perform structural equation modeling while STATA version 14.1 is used for all other analyses. Statistical significance is primarily assessed at the 5% level. However, for examination of moderation using four separate regression models, Bonferroni’s correction is implemented to adjust for multiple comparisons such that statistical significance is assessed at the 1.25% level.

## Results

### Study population characteristics

Table 1 shows the study population characteristics at wave 2 (2004). The mean age is 74 years, and women comprise 55% of respondents. Using the FI, almost 20% of them are classified as being frail at wave 2, with this proportion being higher among women and those aged 75 years and older. This proportion increases to almost 25% at wave 6, with corresponding increase over time observed across gender and age group. Among multidimensional conditions at baseline (wave 2), there are minor gender-specific differences in levels of chronic disease, allostatic load, low physical activity, cognitive impairment, and poor social integration. However, differences are more marked for obesity and depressive symptoms which affect women more. As expected, women report less smoking and alcohol consumption, and better social support, but have lower education and wealth. Those in the older age group have higher levels of chronic disease, allostatic load, depressive symptoms, and cognitive impairment, as well as poorer social integration, while having lower levels of physical activity, educational attainment, and wealth than those younger. For them, smoking is more common while obesity and heavy alcohol intake are less so. They also have better social support.

**Table 1:** Characteristics of English Longitudinal Study of Ageing (ELSA) wave 2 respondents aged 65–89 years included in analyses

Variables	All	By gender		By age group	
		Male	Female	<75 years	≥75 years
General					
Mean age, years (SD)	74.0 (6.3)	73.5 (6.2)	74.3 (6.4)	69.3 (2.8)	80.2 (3.9)
Female, n/N (%)	2568/4638 (55.4)	–	–	1399/2643 (52.9)	1169/1995 (58.6)
Physical frailty					
Mean average walking speed, m/s (SD)	0.8 (0.3)^1^	0.9 (0.3)^2^	0.8 (0.3)^3^	0.9 (0.3)^4^	0.7 (0.3)^5^
Hand grip strength, kg (SD)	25.9 (10.2)^6^	33.4 (8.9)^7^	19.6 (6.1)^8^	28.4 (10.2)^9^	22.2 (8.2)^10^
Exhaustion, n/N (%)	1490/4510 (33.0)	568/1997 (28.4)	922/2513 (36.7)	728/2596 (28.0)	762/1914 (39.8)
Frailty by frailty index, n/N (%)					
Wave 2	717/3647 (19.7)	236/1639 (14.4)	481/2008 (24.0)	322/2207 (14.6)	395/1440 (27.4)
Wave 4	507/2371 (21.4)	158/1051 (15.0)	349/1320 (26.4)	279/1571 (17.8)	228/800 (28.5)
Wave 6	438/1774 (24.7)	145/768 (18.9)	293/1006 (29.1)	285/1325 (21.5)	153/449 (34.1)
Physical					
Obesity, n (%)	1018/3976 (25.6)	400/1783 (22.4)	618/2193 (28.2)	662/2328 (28.4)	356/1648 (21.6)
Mean chronic disease count [out of 14] (SD)	1.9 (1.4)^11^	1.8 (1.4)^12^	2.0 (1.4)^13^	1.8 (1.4)^14^	2.1 (1.5)^15^
Mean allostatic load score [out of 8] (SD)	2.0 (1.5)^16^	1.9 (1.5)^17^	2.1 (1.5)^18^	1.9 (1.5)^19^	2.1 (1.5)^20^
Mean low physical activity level, [0–3] (SD)	1.2 (0.9)^21^	1.1 (0.9)^22^	1.3 (0.9)^23^	1.0 (0.9)^24^	1.4 (0.9)^25^
Smoking history, n (%)	2963/4634 (63.9)	1567/2069 (75.7)	1396/2565 (54.5)	1649/2639 (62.5)	681/1995 (65.9)
Heavy alcohol intake, n (%)	1249/3871 (32.3)	720/1742 (41.3)	529/2129 (24.9)	792/2344 (33.8)	457/1527 (29.9)
Psychological					
Mean CESD-8 score [0–8] (SD)	1.7 (2.0)^26^	1.3 (1.7)^27^	1.9 (2.1)^28^	1.5 (1.9)^29^	1.9 (2.0)^30^
Mean cognitive impairment score [0–49] (SD)	27.5 (6.3)^31^	26.3 (6.4)^32^	25.5 (6.5)^33^	24.1 (6.0)^34^	28.4 (6.3)^35^
Social					
Low education, n (%)	2256/4618 (48.9)	855/2061 (41.5)	1401/2557 (54.8)	1158/2630 (44.0)	1098/1998 (55.2)
Low wealth, n (%)	980/4557 (21.5)	365/2022 (18.1)	615/2535 (24.3)	454/2584 (17.6)	526/1973 (26.7)
Mean poor social support score [0–54] (SD)	13.7 (7.0)^36^	14.7 (7.0)^37^	12.9 (6.8)^38^	13.9 (7.0)^39^	13.3 (6.8)^40^
Mean poor social integration score [0–14] (SD)	6.6 (2.5)^41^	6.7 (2.6)^42^	6.5 (2.5)^43^	6.4 (2.5)^44^	7.0 (2.6)^45^

Unless indicated otherwise, N = 4638 (all), 2070 (male), 2568 (female), 2643 (less than 75 years old), and 1995 (at least 75 years old)

*Frailty* frailty index ≥0.25, *CESD-8* Center for Epidemiologic Studies Depression Scale (8 items)

N = ^1^4096, ^2^1826, ^3^2266, ^4^2400, ^5^1692, ^6^3869, ^7^1760, ^8^2109, ^9^2276, ^10^1593, ^11^4608, ^12^2052, ^13^2556, ^14^2617, ^15^1991, ^16^2319, ^17^1064, ^18^1255, ^19^1436, ^20^883, ^21^4567, ^22^2032, ^23^2535, ^24^2611, ^25^1956, ^26^4479, ^27^1987, ^28^2492, ^29^2586, ^30^1893, ^31^4349, ^32^1946, ^33^2403, ^34^2546, ^35^1803, ^36^3339, ^37^1529, ^38^1810, ^39^2068, ^40^1271, ^41^3267, ^42^1506, ^43^1761, ^44^2035, ^45^1232

Among the performance measures on which the three indicators for physical frailty are based, hand grip strength (weakness) clearly decreases at successive waves across gender and age group, while walking speed (slowness) does so very minimally or not at all. The trends are mixed for exhaustion with either increase or decrease in proportion reporting this across waves (Supplementary Materials, Table 6). Notably, missing values increase to 50–60% by wave 6. In addition, time-varying predictors show increased mean values across waves, with most also doing so across gender and age group (Supplementary Materials, Table 7). Here, missing values occur in 30–40% of respondents by wave 4.

**Fig. 2:**
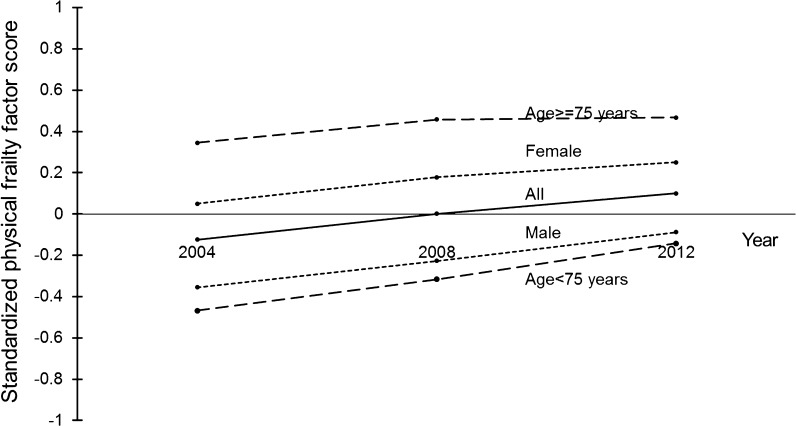
Trajectories of unadjusted physical frailty factor scores across wave 2, 4, and 6 of the English Longitudinal Study of Ageing: mean values for whole group and subgroups. N = 4560 (all), 2025 (male), 2535 (female), 2616 (less than 75 years old), and 1944 (at least 75 years old)

Graphical representation of derived standardized physical frailty factor scores (unadjusted) at waves 2, 4, and 6 is provided in Fig. 2. Over this period, mean differences in standardized physical frailty factor score of individual respondents at wave 6 compared with those at wave 2 for the whole group and subgroups according to gender and age range from 0.12 to 0.33. Although statistically significant (p value less than 0.05) using the dependent samples *t* test (results not shown), these differences are practically small. Mean factor scores for women and those in the older group are higher.

Unique standardized physical frailty factor scores for each respondent at each time point are derived from confirmatory factor analysis using three indicators, namely slowness, weakness, and exhaustion (see “Methods” section).

### Predicted effects

Table 2 shows that even after controlling for the effects of other predictors, older age, female gender, obesity, being underweight, low education, and low wealth are all associated with higher levels of initial physical frailty given their positive and significant coefficients in the first column. On the other hand, smoking is not significantly associated with initial physical frailty, while high alcohol intake has a negative and significant coefficient, and is therefore associated with lower levels of initial physical frailty. Coefficients in the second to fifth columns of Table 2 indicate that the magnitude of effect for obesity is larger among women, while that for low education is larger among men. In addition, the magnitude of effect for older age is larger among those at least 75 years of age, while that for low wealth is larger among those below 75 years of age. However, all these differences across gender and age group are not statistically significant.

**Table 2:** Predictors of initial physical frailty: standardized coefficients of latent growth curve models

	All	Gender		Age	
		Male	Female	<75 years	≥75 years
Effects of time-invariant predictors (wave 2) on physical frailty intercept factor					
Older age	0.563*	0.569*	0.584*	0.207*	0.443*
Female gender	0.419*	–	–	0.449*	0.484*
Obesity	0.101*	0.036	0.152*	0.132*	0.091*
Underweight	0.051*	0.085	0.033	0.064	0.048
Smoking history	0.038	0.032	0.043	0.059*	0.017
High alcohol intake	−0.101*	−0.078*	−0.120*	−0.129*	−0.083*
Low education	0.147*	0.189*	0.116*	0.177*	0.141*
Low wealth	0.113*	0.112*	0.122*	0.163*	0.078*

Standardized coefficients are interpreted as change in physical frailty intercept in standard deviation (SD) units for a one SD increase in continuous predictors, or from zero to one for binary predictors (female gender, obesity, underweight, smoking history, high alcohol intake, low education, and low wealth)

N = 4638 (all), 2070 (male), 2568 (female), 2643 (less than 75 years old), and 1995 (at least 75 years old)

* Indicates p-value <0.05

Associations with future physical frailty across waves 2, 4, and 6 better reflect their true predictive effects. Firstly, the correlation between the intercept (initial physical frailty) and slope (physical frailty change) factors is −0.206 (p-value >0.05), indicating that a non-significant trend towards higher levels of initial physical frailty is associated with less steep increase in physical frailty over time. This could be related in part to a ceiling effect. Next, among the time-invariant predictors, none predict greater increase in physical frailty levels over time, controlling for the effects of other predictors, given the non-significant coefficients in the first column in the upper section of Table 3. However, the predictive effect of older age is stronger and significant in men and those less than 75 years of age, although differences across gender and age group are not statistically significant. Among time-varying predictors, chronic disease, allostatic load, low physical activity, depressive symptoms, cognitive impairment, and poor social support all predict higher future physical frailty levels controlling for the effects of other time-varying predictors as well as those of time-invariant predictors on the physical frailty slope factor. The statistically significant coefficients in the first column in the lower section of Table 3 indicate that one SD increase in levels of these conditions predicts increase of 0.07–0.24 SD in physical frailty levels 2 years later. These are non-trivial effects given that the mean physical frailty level of the study population only increases by approximately 0.06 SD over 2 years. Judging by the coefficients in the second to fifth columns, the magnitude of effect is generally consistent across gender and age group with the exception of those for depressive symptoms and poor social support which are higher in the older age group, although these differences are not significant. Notably, poor social integration did not predict higher physical frailty levels.

**Table 3:** Predictors of future physical frailty (waves 2, 4, and 6): standardized coefficients from latent growth curve models

	All	Gender		Age	
		Male	Female	<75 years	≥75 years
Effects of time-invariant predictors (wave 2) on physical frailty slope factor					
Older age	0.288	0.481*	0.132	0.226*	−0.071
Female gender	0.062	–	–	0.294	−0.560
Obesity	0.156	0.210	0.114	0.104	0.214
Underweight	−0.040	<0.001	−0.063	−0.058	0.029
Smoking history	−0.058	−0.028	−0.089	−0.074	−0.003
High alcohol intake	0.019	−0.010	0.047	0.073	−0.101
Low education	−0.058	0.077	−0.139	−0.055	−0.030
Low wealth	0.100	−0.039	0.174	0.090	−0.051
Effects of lagged time-varying predictors (waves 1, 2, and 4) on physical frailty factor (waves 2, 4, and 6)					
Chronic disease	0.236*	0.264*	0.220*	0.259*	0.271*
Allostatic load	0.108*	0.132*	0.088*	0.118*	0.130*
Low physical activity	0.189*	0.191*	0.193*	0.205*	0.192*
Depressive symptoms	0.115*	0.130*	0.108*	0.108*	0.167*
Cognitive impairment	0.182*	0.222*	0.160*	0.181*	0.195*
Poor social support	0.067*	0.065*	0.074*	0.063*	0.109*
Poor social integration	0.007	0.029	−0.015	0.016	−0.024

For time-invariant predictors, standardized coefficients are interpreted as change in physical frailty slope in standard deviation (SD) units for one SD increase in continuous predictors, or from zero to one for binary predictors (female gender, obesity, underweight, smoking history, high alcohol intake, low education, and low wealth). For time-varying predictors, standardized coefficients are interpreted as increase in physical frailty factor in SD units for their one SD increase

N = 4638 (all), 2070 (male), 2568 (female), 2643 (less than 75 years old), and 1995 (at least 75 years old)

* Indicates p-value <0.05

### Moderated and mediated effects

Beyond gender- and age group-specific effects observed, moderated effects of predictors across specific subgroups are shown in Table 4. Among time-invariant predictors, female gender has a stronger effect on physical frailty change for those with poorer social support and poorer social integration, while obesity has a stronger effect on physical frailty change for those with lower physical activity, poorer social support, and poorer social integration. Among time-varying predictors, allostatic load has a stronger effect on future physical frailty for those with more depressive symptoms and poorer social integration, while low physical activity has a stronger effect for those with poorer social support. However, all these differences do not reach statistical significant levels.

**Table 4:** Moderation of predictors of future physical frailty: standardized coefficients from latent growth curve models

	Low physical activity		Depressive symptoms		Poor social support		Poor social integration	
	Below mean^a^	Above mean^b^	Below mean^c^	Above mean^d^	Below mean^e^	Above mean^f^	Below mean^g^	Above mean^h^
Effects of time-invariant predictors (wave 2) on physical frailty slope factor								
Older age	0.324**	0.117	0.246*	0.157	0.342*	0.198	0.514**	0.285
Female gender	0.171	−0.174	0.106	0.012	−0.139	0.262	0.002	0.179
Obesity	0.079	0.199*	0.112	0.112	0.026	0.220	0.069	0.407*
Underweight	−0.131	0.197	−0.081	0.041	−0.112	0.021	−0.110	0.047
Smoking history	−0.001	−0.087	−0.046	0.042	−0.091	−0.021	−0.204	0.046
High alcohol intake	−0.045	0.138	−0.014	0.093	0.002	0.020	0.009	0.054
Low education	−0.063	0.029	−0.018	−0.038	−0.047	−0.024	−0.014	−0.156
Low wealth	0.171*	−0.029	0.094	0.048	0.162	0.024	0.233	0.101
Effects of lagged time-varying predictors (waves 1, 2, and 4) on physical frailty factor (waves 2, 4, and 6)								
Chronic disease	0.233**	0.243**	0.240**	0.229**	0.261**	0.216**	0.247**	0.222**
Allostatic load	0.078**	0.099**	0.108**	0.135**	0.109**	0.109**	0.095**	0.121**
Low physical activity	0.132**	0.140**	0.185**	0.189**	0.164**	0.208**	0.176**	0.191**
Depressive symptoms	0.098**	0.120**	0.054**	0.038*	0.127**	0.101**	0.111**	0.122**
Cognitive impairment	0.177**	0.207**	0.203**	0.164**	0.187**	0.181**	0.181**	0.180**
Poor social support	0.091**	0.058*	0.071**	0.009	0.068**	0.012	0.059**	0.072**
Poor social integration	−0.015	0.032	0.005	0.008	0.003	0.010	0.019	−0.011

N = ^a^2819, ^b^1819, ^c^3324, ^d^1314, ^e^2275, ^f^2363, ^g^2244, ^h^2394

For time-invariant predictors, standardized coefficients are interpreted as change in physical frailty slope in standard deviation (SD) units for one SD increase in continuous predictors, or from zero to one for binary predictors (female gender, obesity, underweight, smoking history, high alcohol intake, low education, and low wealth). For time-varying predictors, standardized coefficients are interpreted as increase in physical frailty factor in SD units for their one SD increase

* Indicates p-value <0.05 but ≥0.0125

** Indicates p-value <0.0125 (to take into account Bonferroni’s correction for 4 comparison models)

Indirect or mediated effects of time-varying predictors on physical frailty slope factor are shown in Table 5. Among these, the indirect effects of low physical activity, depressive symptoms, and cognitive impairment on future physical frailty through chronic disease and allostatic load are significant, given their respective coefficients in the first column. Indirect effects through chronic disease are stronger than those through allostatic load. Together, they account for at most one-fifth of the total effects of these predictors (results not shown). There are minor and non-significant differences in indirect effects through chronic disease and allostatic load across gender and age group.

**Table 5:** Effects of predictors (waves 1, 2, and 4) on future physical frailty (waves 2, 4, and 6) mediated by chronic disease and allostatic load (waves 2, 4, and 6): standardized coefficients from latent growth curve models

	All	Gender		Age	
		Male	Female	<75 years	≥75 years
Indirect effect on physical frailty through chronic disease					
Low physical activity	0.052*	0.047*	0.054*	0.048*	0.065*
Depressive symptoms	0.036*	0.041*	0.029*	0.038*	0.041*
Cognitive impairment	0.015*	0.020*	0.014*	0.017*	0.004
Poor social support	0.007	0.004	0.013*	0.013*	0.001
Poor social integration	−0.004	−0.001	−0.008	−0.003	−0.012
Indirect effect on physical frailty through allostatic load					
Low physical activity	0.007*	0.009*	0.004	0.009*	0.004*
Depressive symptoms	0.002*	0.008*	<0.001	0.002*	0.004*
Cognitive impairment	0.004*	0.002*	0.003	0.003*	0.006
Poor social support	0.001	0.003	0.001	0.003	−0.003
Poor social integration	0.001	0.002	<0.001	<0.001	0.002

Standardized coefficients are interpreted as increase in physical frailty factor in SD units for one SD increase in the predictors

N = 4638 (all), 2070 (male), 2568 (female), 2643 (less than 75 years old), and 1995 (at least 75 years old)

* Indicates p-value < 0.05

The results for moderation of indirect effects are provided in Supplementary Materials (Table 8). Overall, there are minor and non-significant differences in indirect effects across categories of low physical activity, depressive symptoms, poor social support, and poor social integration. The exception is the stronger indirect effect of poor social support through chronic disease among those with poorer social integration, with the difference being statistically significant at the 5%, but not 1.25% level.

### Sensitivity analyses

Sensitivity analyses that explore MNAR by implementing the Wu and Carroll selection model for Model 1 indicate that coefficients are only trivially different from those assuming MAR using FIML (results not shown). In other words, assuming the worst case scenario that missing values due to dropout by death or other reasons are MNAR does not change the interpretation of the results. Furthermore, specifying depressive symptoms with the full set of eight items of the CESD instrument rather than just six of them as we did only results in marginal changes in the coefficient for depressive symptoms (results not shown). It is also worth mentioning that most of the key findings on moderation are significant when accounting for multiple comparisons with Bonferroni’s correction.

## Discussion

Among ELSA respondents, we find evidence that chronic disease, allostatic load, low physical activity, depressive symptoms, cognitive impairment, and poor social support all predict increase in future physical frailty levels after accounting for the effects of other measured predictors. In other words, these predictors adversely influence the trajectory of physical frailty over time, assuming that the physical, psychological, and social predictors we controlled for in our analyses are sufficient to account for important confounding due to omitted variables. In general, our findings are consistent with those of previous studies using of the broader CHS frailty phenotype with all five indicators (Gruenewald et al. 2009; Ottenbacher et al. 2009; Peek et al. 2012; Syddall et al. 2010; Woods et al. 2005) or an even broader multidimensional frailty specification (Strawbridge et al. 1998). However, we did not observe that female gender, obesity, underweight, smoking, high alcohol intake, low education level, low wealth, and poor social integration influence physical frailty progression as suggested by previous studies (Etman et al. 2012; Gruenewald et al. 2009; Peek et al. 2012; Strawbridge et al. 1998; Syddall et al. 2010; Woods et al. 2005). A possible explanation is that we use a narrower physical frailty specification. Moreover, compared with the aforementioned studies, our analyses included adjustment for a wider set of potential confounders. In addition, it is possible that female gender, low education, and low wealth may have already exerted a major part of their effects on initial physical frailty, and thus may not have any additional and significant impact during the follow-up years of our study. Furthermore, the effects of other predictors such as obesity, underweight, and poor social integration may overlap with those of stronger predictors and be subsumed under the effects of the latter. Finally, our choice for operationalization of these predictors may not be optimal with respect to representing the intended constructs, thereby resulting in attenuation of any true effects.

On the other hand, the association of obesity, low education, and low wealth with initial physical frailty may to an extent reflect prior health and social conditions in early to mid-life. Thus, these conditions can arguably be considered predictors of initial physical frailty observed in our study. In the case of smoking, its association with initial physical frailty may be attenuated and therefore, not significant due to selection effects in that smokers with more adverse health may have died and are not available for inclusion in the study at wave 2. The negative association between high alcohol intake and initial physical frailty may be explained by reverse causality where people with higher frailty levels are likely to consume less alcohol by reason of their ill health.

We could not demonstrate any significant gender- or age-specific effects of predictors of physical frailty. In addition, we do not find evidence of moderation by low physical activity, depressive symptoms, poor social support, and poor social integration, because observed differences in effects of predictors are not statistically significant across categories of these four conditions.

However, we identify chronic disease and allostatic load as mediators of indirect effects on physical frailty, albeit only for selected predictors, namely low physical activity, cognitive impairment, and depressive symptoms. To date, similar findings have not been reported. These findings answer in part the question on *how* predictors exert their effects. Another point worth highlighting is that we have restricted the choice of candidate mediators to those identified by the Canadian working framework (Bergman et al. 2004). It is quite possible that other lifestyle-related and psychological conditions may be mediators. Finally, we demonstrate the moderating effect of poor social integration on the indirect effect of poor social support through chronic disease, which reflects the role of social conditions on pathways to physical frailty. To a limited extent, this finding answers the question of *for whom* the indirect effect of predictors of physical frailty is stronger.

While the predictors we identified were shown to be associated with future frailty, either singly or in various combinations by separate previous studies, their roles together as predictors is demonstrated in our study while controlling for a broader range of multidimensional conditions. Thus, our findings are likely to be more robust to bias arising from unmeasured confounding than those of these previous studies. To our knowledge, this is also the first report on mediators of predictors of future physical frailty. Overall, our findings contribute to further understanding of development of physical frailty in older people by providing a set of key pathways on which to build upon in future research.

Beyond this, our findings are also relevant to health and social policy formulation. Particularly, knowledge of predictors of physical frailty progression as well as their mediators and moderators informs thinking on how physical frailty may be potentially modified by interventions. Based on our findings, chronic disease, allostatic load, low physical activity, depressive symptoms, cognitive impairment, poor social support, and poor social integration represent target conditions for programs and policies directed at reducing physical frailty in older people. Moreover, obesity, low education, and low wealth represent prior conditions which could be better addressed in young and middle-aged people in the hope of reducing the risk of developing physical frailty as they transit to later life. While health and social care initiatives to address some of these issues may already exist in certain jurisdictions, focus on addressing specific components of allostatic load have to date received less attention. For example, reducing chronic systemic inflammation from early life through lifestyle changes in diet, weight loss, and exercise is a specific area for attention (Nicklas et al. 2005). Equally important, population-level initiatives to identify depression and facilitate or encourage physical activity may need drawing up or bear strengthening if already in place. Poor social support is a more challenging issue at it occurs at the personal relationship level. Public education highlighting the importance of social support, and particularly that of providing emotional support should be explored. Poor social integration may be addressed by provision of interventions designed to reduce social isolation including social facilitation interventions involving group-based activities such as friendship clubs, day care centers, and social networking, Other potentially useful interventions include community gatekeepers, geriatric rehabilitation, visitation programs, as well as leisure and skill development activities such as gardening, computer use, and voluntary work (Gardiner et al. 2016).

Although informative, our findings nevertheless point to specific gaps in the understanding of physical frailty in older people. To begin with, further research to identify specific subgroups for whom the predictive effects on physical frailty are stronger is needed. Psychological deficits and adverse social conditions may define these subgroups. Finally, and as alluded to earlier, the possibility of alternative mediators including psychological conditions such as depression should be explored.

From a methodological perspective, our study has a number of important limitations. Firstly, this is an observational investigation using secondary data. This imposes limits to which we are able to specify predictors, especially those in the psychological and social domains. However, using the available data, we are able to operationalize established measurement instruments such as CESD for depressive symptoms, and implement composite measures devised by others to represent more complex constructs such as poor social support and poor social integration (Banks et al. 2010). Beyond measurement, unobserved confounding due to omitted variables may introduce bias. To address this in our analyses, we include a broad set of important multidimensional predictors which control for each other. However, genetic influences and childhood social conditions are not included. Although ELSA includes a life history interview conducted at wave 3, information on adverse circumstances in childhood is unavailable for about half of our study population due to death or attrition by then. Of interest, childhood socioeconomic position was found to be associated with relatively small reductions in gait speed and grip strength (Birnie et al. 2011). Nevertheless, unless the effects of omitted variables such as these are large and highly correlated with those of other predictors, it is not very likely that any residual confounding will be severe enough to alter our study conclusions. Secondly, missing values which are inevitable in a longitudinal study such as ours pose threats to validity. These are handled by FIML which assumes that missing values are MAR. However, missing values due to dropout or death may be MNAR, given that their occurrence may be conditional on prior values of physical frailty. Thus, we incorporate more advanced selection models that account for missing values which may be MNAR. Indeed, these sensitivity analyses do not change the main results more than trivially, thus providing some reassurance that our study conclusions are robust to missing values. Finally, our use of separate models for estimating moderating effects increases the risk of discovering significant effects purely by chance. To mitigate this risk, we restrict our analyses to those investigating a limited set of pathways that are defined a priori, and use Bonferroni’s correction to account for multiple comparisons. Applying the latter procedure, most of our key results remain statistically significant. Overall, although we cannot assume causation from statistical association, biological plausibility and consistency with previous studies strengthen our key findings.

In conclusion, our study validates at least in part the pathways to frailty put forth by the Canadian working framework (Bergman et al. 2004). Potentially modifiable predictors of future physical frailty in late life extend across more than one domain, and include low physical activity, cognitive impairment, depressive symptoms, and poor social support. In addition, obesity, low education, and low wealth may be addressable early or mid-life predictors. Moreover, chronic disease and allostatic load are mediators, while poor social integration is a moderator on pathways to physical frailty. These findings provide supporting evidence for multi-pronged population-level health and social interventions that target these conditions in broad strategies for minimizing physical frailty in older people.

## Electronic supplementary material

Below is the link to the electronic supplementary material.

Supplementary material 1 (DOCX 313 kb)
