# Supplementary material for: Multidimensional predictors of physical frailty in older people: identifying how and for whom they exert their effects
Source: Biogerontology. 2017 Feb 3;18(2):237–52. doi: 10.1007/s10522-017-9677-9 (PMC5350240; doi:10.1007/s10522-017-9677-9)
Supplement: Supplementary file 1 — Supplementary material 1 (DOCX 313 kb) [file 10522_2017_9677_MOESM1_ESM.docx]

**Supplementary Material**

**Scoring systems of key variables**

**Chronic disease**

| **Condition** | **Definition** |
| --- | --- |
| Hypertension (High blood pressure) | Whether ever reported high blood pressure |
| Angina | Whether ever reported angina |
| Myocardial Infarction (Heart attack) | Whether ever reported myocardial infarction |
| Congestive Heart Failure | Whether ever reported congestive heart failure |
| Arrhythmia (Abnormal heart rhythm) | Whether ever reported arrhythmia |
| Diabetes Mellitus | Whether ever reported diabetes |
| Stroke | Whether ever reported stroke |
| Asthma | Whether ever reported asthma |
| Arthritis | Whether ever reported arthritis |
| Osteoporosis | Whether ever reported osteoporosis |
| Cancer | Whether ever reported cancer |
| Parkinson’s Disease | Whether ever reported Parkinson’s Disease |
| Psychiatric disorders | Whether ever reported psychiatric disorders |
| Dementia | Whether ever reported dementia |

Note: Presence of each comorbid condition is assigned a score of 1. The total score is from 0 to 14.

**Allostatic load**

Allostatic load is the physiological dysregulation in multiple body systems and is specified by nine biomarkers including blood pressure readings, anthropometric measurements, and blood tests for cholesterol levels, glucose control, and inflammatory markers. These biomarkers are:

| **Biomarker** | **Definition** |
| --- | --- |
| Systolic blood pressure | whether >150 mmHg or not |
| Diastolic blood pressure | whether >80 mmHg or not |
| Glycosylated haemoglobin level | whether >5.8% or not |
| Serum triglyceride level | whether >2.2 mmol/l or not |
| Serum c-reactive protein level | whether >4.7 mg/l or not |
| Serum fibrinogen level | whether >3.7 umol/l or not |
| Peak expiratory flow rate | whether <232 l/min or not |
| Waist-hip ratio | whether >0.9588534 or not |

Note: For each biomarker, a score of one is awarded for values beyond a cut-off level reflecting high risk (75^th^ percentile), with a score of zero given if otherwise.

**Poor social integration**

| **Item** | **Definition** |
| --- | --- |
| Living with spouse for partner | Whether having no spouse or partner living with them (1) |
| Little contact with children | Whether contact by meeting, phoning, or email is:  - at least once per week (0)  - once or twice a month (1)  - once every few months (2)  - once or twice a year or less (3) |
| Little contact with other family members | Whether contact by meeting, phoning, or email is:  - at least once per week (0)  - once or twice a month (1)  - once every few months (2)  - once or twice a year or less (3) |
| Little contact with friends | Whether contact by meeting, phoning, or email is:  - at least once per week (0)  - once or twice a month (1)  - once every few months (2)  - once or twice a year or less (3) |
| Low membership of non-religious organizations, clubs or societies | Whether having membership of the following groups or organizations (number):  1) Political party, trade union or environmental groups  2) Tenants groups, resident groups, neighbourhood watch  3) Charitable associations  4) Education, arts or music groups or evening classes  5) Social Clubs  6) Sports clubs, gyms, exercise classes  - 0 (0)  - 1 or 2 (1)  - 3 or 4 (2)  - 5 or 6 (3) |
| Not a member of a religious group | Whether not a member of any church or other religious group (1) |

Note: Individual scores for each item are indicated in brackets. The total score (0 to 14) is a measure of the extent of social isolation.

**Poor social support**

| **Item** | **Definition** |
| --- | --- |
| Lack of positive support | Answers to questions on whether children:  “understand the way you feel”   - a lot (0) - some (1) - a little (2) - not at all (3)   “can rely on if you had a serious problem”   - a lot (0) - some (1) - a little (2) - not at all (3)   “can open up to them if you need to talk”   - a lot (0) - some (1) - a little (2) - not at all (3)   In turn, answers for these questions are also sought with respect to other family members, and friends.  Total score: 0 to 27 |
| Negative support | Answers to questions on whether children:  "criticizes the respondent"   - a lot (3) - some (2) - a little (1) - not at all (0)   "lets the respondent down"   - a lot (3) - some (2) - a little (1) - not at all (0)   "gets on the nerves of respondent"   - a lot (3) - some (2) - a little (1) - not at all (0)   In turn, answers for these questions are also sought with respect to other family members, and friends.  Total score: 0 to 27 |

Note: Individual scores for each item are indicated in brackets. The total score (0 to 54) is a measure of the extent of deficient emotional support or negative social interaction.

**Frailty Index (30 items)**

| **Item** | **Definition** |
| --- | --- |
| Chronic illness: Hypertension | Whether ever reported high blood pressure |
| Chronic illness: Myocardial Infarction | Whether ever reported myocardial infarction |
| Chronic illness: Congestive heart failure | Whether ever reported congestive heart failure |
| Chronic illness: Diabetes Mellitus | Whether ever reported diabetes |
| Chronic illness: Stroke | Whether ever reported stroke |
| Chronic illness: Arthritis | Whether ever reported arthritis |
| Chronic illness: Cancer | Whether ever reported cancer |
| Chronic illness: Obesity | BMI: WHO definition of obesity (>=30) or underweight (<18.5) |
| Psychological condition: Dementia | Whether ever reported dementia |
| Psychological condition: Feeling depressed (CESD) | Whether…felt depressed much of the time during the past week |
| Psychological condition: Feeling effortful (CESD) | Whether…felt everything they did during the past week was an effort |
| Psychological condition: Feeling happy (CESD): reverse | Whether…happy much of the time during the past week (reverse) |
| Psychological condition: Feeling lonely (CESD) | Whether…felt lonely much of the time during the past week |
| Psychological condition: Could not get going (CESD) | Whether…could not get going much of the time during the past week |
| Poor self-rated health | Whether self-rated health reported as “poor” |
| Mobility: walking 100 yards | Difficulty walking 100 yards |
| Mobility: getting up from chair | Difficulty getting up from chair after sitting long periods |
| Mobility: climbing stairs | Difficulty climbing one flight stairs without resting |
| Mobility: lifting weights | Difficulty lifting or carrying weights over 10 pounds |
| BADL: dressing | Difficulty dressing, including putting on shoes and socks |
| BADL: walking | Difficulty walking across a room |
| BADL: bathing | Difficulty bathing or showering |
| BADL: eating | Difficulty eating, such as cutting up food |
| BADL: toileting | Difficulty using the toilet, including getting up or down |
| IADL: shopping | Difficulty shopping for groceries |
| IADL: taking medication | Difficulty taking medications |
| IADL: doing housework | Difficulty doing work around house and garden |
| IADL: managing finances | Difficulty managing money, eg paying bills, keeping track expenses |
| Weak grip strength | Weak grip strength (1st measurement dominant hand) using gender-specific cut-offs (male: <30kg female: <20 kg) |

Note: Positive response or finding for each item is assigned a score of 1. The total score is divided by 30 to obtain the frailty Index (0 to 1).

**Mathematical equations for the statistical models**

Let *y_ti_* denote physical frailty for individuals *i* = 1..., *n* at times *t* = 0, 1, and 2 corresponding to waves 2, 4, and 6 respectively, and let *x_i_* be a vector of time-invariant predictors and *w_t-1i_* be a vector of lagged time-varying predictors for *y_ti_*.

The latent growth curve model for *y_ti_* (Model 1) is

*y_ti_* = *η*_0_*_i_* + *η*_1_*_i_t* + *γw_t-_*_1_*_i_* + *ε_ti_*

for subject *i* at times *t* = 0, 1, and 2, where *ε_ti_* is a normally distributed residual with mean 0 and variance *σ_ε_*, and where

*η*_0_*_i_* = *α*_0_ + *β*_0_*x_i_* + *ζ*_0_*_i_* (a)

*η*_1_*_i_* = *α*_1_ + *β*_1_*x_i_* + *ζ*_1_*_i_*  (b)

are referred to as the intercept growth factor and the slope growth factor (i.e. the coefficient of time *t*) respectively, and where *ζ*_0_*_i_* and *ζ*_1_*_i_* are normally distributed random effects with means 0 and variances *σ_ζ_*_0_ and *σ_ζ_*_1_ and covariance *σ_ζ_*_01_.

Here, the coefficients *β*_0_ describe the associations between the time-invariant predictors and physical frailty at wave *t* = 0, the coefficients *β*_1_ the effect of time-invariant predictors on the coefficient of *t* on physical frailty (the time slope), and the coefficients *γ* the association between time-varying predictors and within-person change in physical frailty, The estimated coefficients *β*_0_, *β*_1_, and *γ* for different predictors are shown in Table 2, and the upper and lower parts of Table 3 respectively.

To estimate gender- and age-specific effects, we use the same latent growth curve model for *y_ti_*, but stratified into two subgroups according to gender and age group.

**Moderation.** Here, we use the latent growth curve model for *y_ti_* (Model 1) again, but stratified into two subgroups according to four moderating variables, namely low physical activity, depressive symptoms, poor social support, and poor social integration (Model 2).

**Mediation.** Here we use the results of standard linear path analysis, applied to model (b) for the slope factor. For simplicity of notation in introducing the idea, consider the case of two lagged time-varying predictors *w_t-_*_1_*_i_* = (*v_t-_*_1_*_i_*, *m_t-_*_1_*_i_*) (the extension to cases with more variables is also analogous), the latent growth curve model for y_ti_ is then

*y_ti_* = *η_0i_* + *η*_1_*_i_t* + *γ*_1_*v_t-_*_1_*_i_* + *γ*_2_*m_t-_*_1_*_i_* + *ε_ti_*

Again, suppose further that

*m_t-_*_1_*_i_* = *λ*_0_ + *λ*_1_*v_t-_*_1_*_i_* + *δ_t-_*_1_*_i_*.

Then the model given *v_t-1i_* only, averaging over the distribution of *m_t-_*_1_*_i_*, is

*y_ti_* = *η*_0_*_i_* + *η*_1_*_i_t* + *γ_*1_v_t-_*_1_*_i_* + *ε_*ti_*

where *ε_*ti_* = (*ε_ti_* + *γ*_2_*δ_t-_*_1_*_i_*) and *γ^∗^*_1_ = *γ*_1_ + *γ*_2_*λ*_1_. Here *γ^∗^*_1_ is the total effect of the variable *v_t-_*_1_*_i_* on *y_ti_*, *γ*_1_ is the direct effect of *v_t-_*_1_*_i_*, and *γ*_2_*λ*_1_ the indirect effect of *v_t-_*_1_*_i_* mediated via *m_i_* (Model 3).

Here, to estimate gender- and age-specific effects, we use the same mediation model, but stratified into two subgroups according to gender and age group.

**Moderated mediation.** Here, we use the latent growth curve model for *y_ti_* including mediated effects (Model 3) but stratified into two subgroups according for gender, age group, and four moderating variables, namely low physical activity, depressive symptoms, negative social interactions, and weak social network.

Here, we use the same mediation model (Model 3) again, but stratified into two subgroups according to four moderating variables, namely low physical activity, depressive symptoms, poor social support, and poor social integration (Model 4).

**Figure 3. Path diagram of Model 1: conditional latent growth curve model with time-invariant and time-varying predictors**


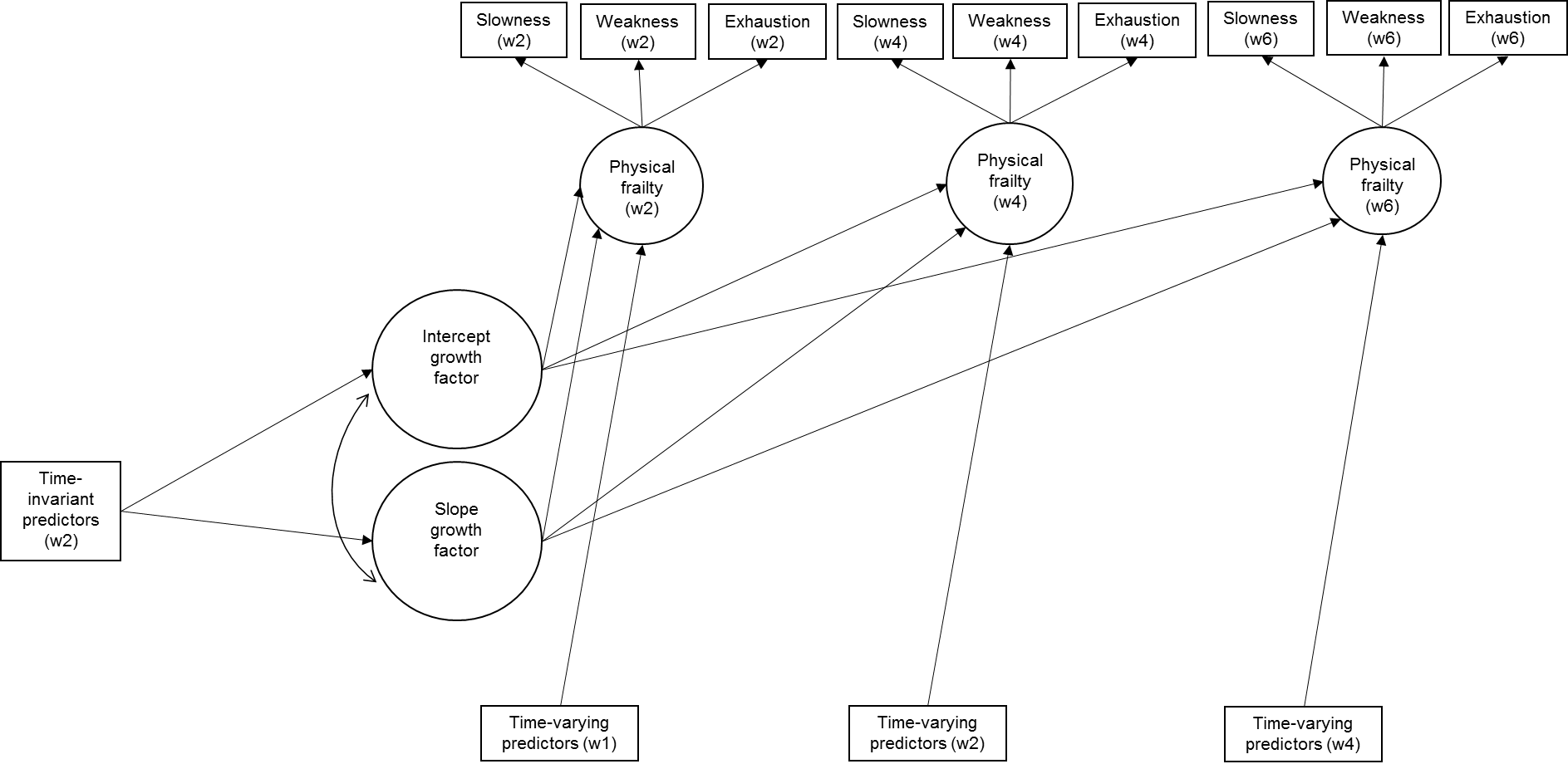


w1: wave 1 w2: wave 2 w4: wave 4 w6: wave 6

Circle: latent variable

Rectangle: observed variable

Single-headed straight arrow: effect of one variable on another

Double-headed curved arrow: covariance between two variables

**Figure 4. Path diagram of Model 3: conditional latent growth curve model with time-invariant and time-varying predictors, and time-varying mediators**


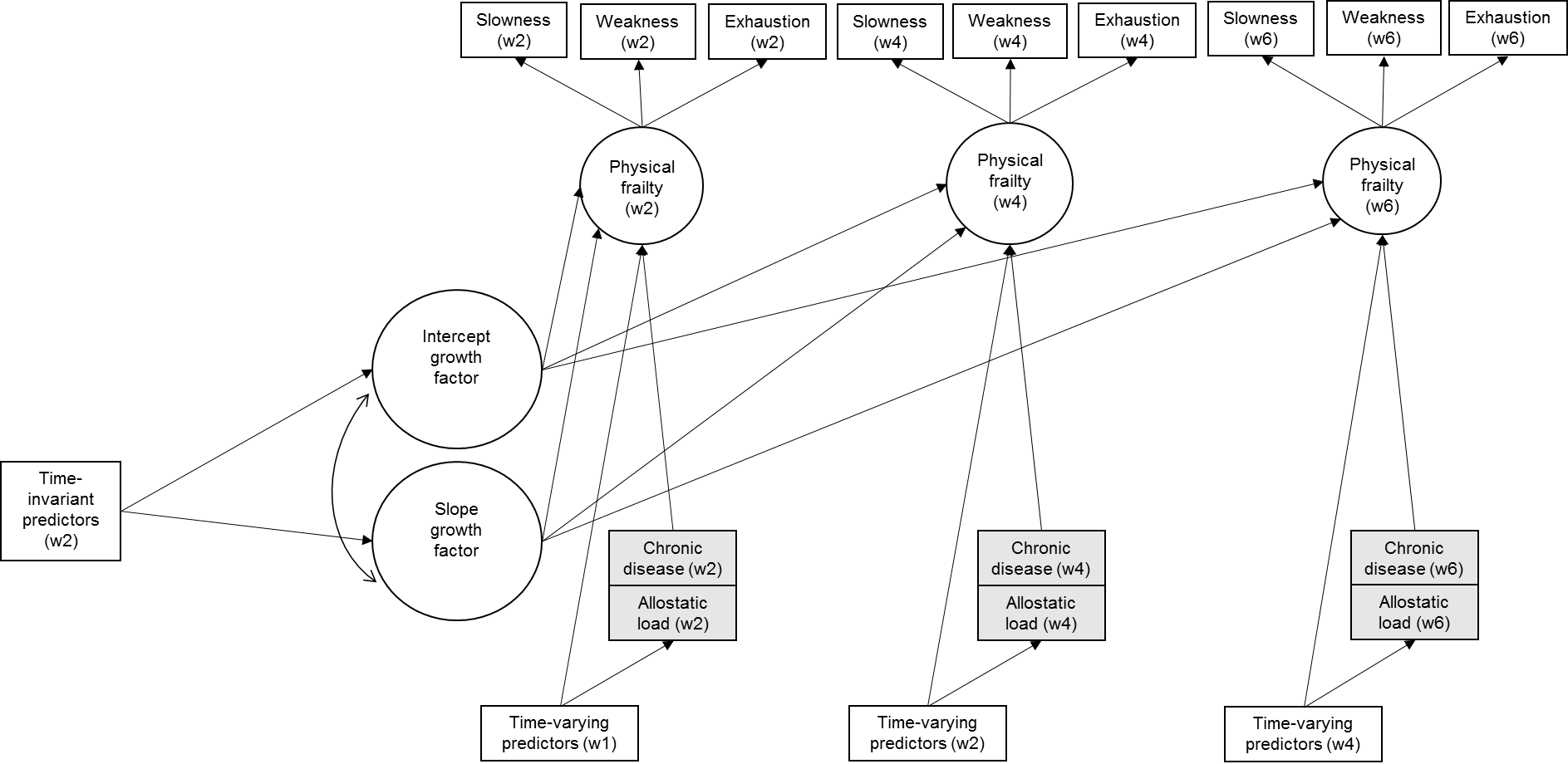


w1: wave 1 w2: wave 2 w4: wave 4 w6: wave 6

Circle: latent variable

Rectangle: observed variable

Single-headed straight arrow: effect of one variable on another

Double-headed curved arrow: covariance between two variables

**Figure 5. Path diagram of Model 1: conditional latent growth curve model with time-invariant and time-varying predictors, and incorporating Wu and Carroll selection model (Enders, 2011) to handle missing not at random (MNAR) data**


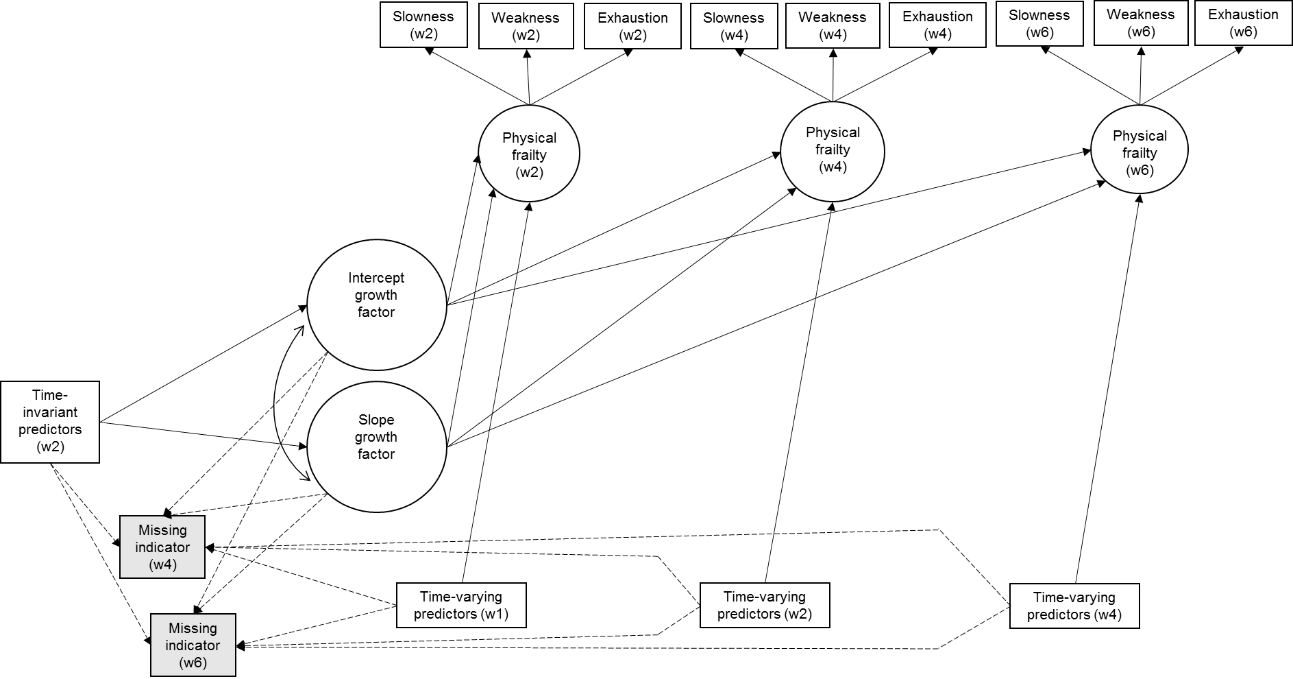


w1: wave 1 w2: wave 2 w4: wave 4 w6: wave 6

Circle: latent variable

Rectangle: observed variable

Single-headed straight arrow: effect of one variable on another

Double-headed curved arrow: covariance between two variables

Single-headed straight arrow (dashed): effect of one variable on another (for Wu and Carroll selection model)

**Table 6. Characteristics of English Longitudinal Study of Ageing (ELSA) wave 2 respondents aged 65 to 89 years included in analyses: physical frailty-related variables (waves 2, 4, and 6)**

| **Variables** | **All** | **By gender** | | **By Age group** | |
| --- | --- | --- | --- | --- | --- |
|  |  | **Male** | **Female** | **< 75 years** | **>= 75 years** |
| **Physical frailty**  Mean average walking speed, m/sec (SD):  Wave 2  Wave 4  Wave 6  Hand grip strength,  kg (SD): Wave 2  Wave 4  Wave 6  Exhaustion, n/N (%):  Wave 2  Wave 4  Wave 6  Frailty by Frailty Index, (SD): Wave 2  Wave 4  Wave 6 | 0.8 (0.3)^1^  0.8 (0.3)^6^  0.8 (0.3)^11^  25.9 (10.2)^16^  24.3 (10.2)^21^  22.8 (9.5)^26^  1,490/4,510 (33.0)  955/2,977 (32.1)  632/1,962 (32.2)  0.15 (0.13)^31^  0.16 (0.13)^36^  0.17 (0.14)^41^ | 0.9 (0.3)^2^  0.8 (0.3)^7^  0.8 (0.3)^12^  33.4 (8.9)^17^  32.0 (9.0)^22^  29.6 (8.8)^27^  568/1,997 (28.4)  327/1,290 (25.4)  218/848 (25.7)  0.13 (0.12)^32^  0.13 (0.11)^37^  0.15 (0.14)^42^ | 0.8 (0.3)^3^  0.7 (0.3)^8^  0.7 (0.3)^13^  19.6 (6.1)^18^  18.2 (6.2)^23^  17.5 (5.9)^28^  922/2,513 (36.7)  628/1,687 (37.2)  414/1,114 (37.2)  0.17 (0.14)^33^  0.18 (0.14)^38^  0.19 (0.14)^43^ | 0.9 (0.3)^4^  0.8 (0.3)^9^  0.8 (0.3)^14^  28.4 (10.2)^19^  26.6 (10.3)^24^  24.4 (9.6)^29^  728/2,596  (28.0)  518/1,868 (27.7)  401/1,402 (28.6)  0.13 (0.12)^34^  0.14 (0.12)^39^  0.16 (0.14)^44^ | 0.7 (0.3)^5^  0.7 (0.2)^10^  0.6 (0.2)^15^  22.2 (9.0)^20^  20.4 (8.6)^25^  18.9 (7.9)^30^  762/1,914  (39.8)  437/1,109 (39.4)  231/560 (41.3)  0.18 (0.14)^35^  0.19 (0.14)^40^  0.21 (0.14)^45^ |

*Note*

Frailty: Frailty Index >=0.25

N = ^1^4,096 ^2^1,826 ^3^2,266 ^4^2,400 ^5^1,692 ^6^2,649 ^7^1,182 ^8^1,467 ^9^1,705 ^10^944 ^11^1,688 ^12^754 ^13^934 ^14^1,254 ^15^434 ^16^3,869 ^17^1,760 ^18^2,109 ^19^2,276 ^20^1,593 ^21^2,531 ^22^1,115 ^23^1,416 ^24^1,621 ^25^910 ^26^1,868 ^27^820 ^28^1,048 ^29^1,339 ^30^529 ^31^3,647 ^32^1,639 ^33^2,008 ^34^2,207 ^35^1,440 ^36^2,371 ^37^1,051 ^38^1,320 ^39^1,571 ^40^800 ^41^1,774 ^42^768 ^43^1,006 ^34^1,325 ^35^449

**Table 7. Characteristics of English Longitudinal Study of Ageing (ELSA) wave 2 respondents aged 65 to 89 years included in analyses: time varying predictors and mediators across waves**

| Variables | All | By gender | | By Age group | |
| --- | --- | --- | --- | --- | --- |
|  |  | Male | Female | < 75 years | >= 75 years |
| **Physical**  Mean chronic disease count [out of 14] (SD):  Wave 2  Wave 4  Wave 6  Mean allostatic load score [out of 8] (SD):  Wave 2  Wave 4  Wave 6  Mean low physical activity level, n (%):  Wave 1  Wave 2  Wave 4 | 1.9 (1.4)^1^  2.2 (1.5)^6^  2.1 (1.5)^11^  2.0 (1.5)^16^  2.2 (1.5)^21^  2.6 (1.2)^26^  1.1 (0.9)^31^  1.2 (0.9)^36^  1.3 (1.0)^41^ | 1.8 (1.4)^2^  2.1 (1.5)^7^  1.9 (1.4)^12^  1.9 (1.5)^17^  2.0 (1.4)^22^  2.5 (1.2)^27^  1.0 (0.9)^32^  1.1 (0.9)^37^  1.2 (1.0)^42^ | 2.0 (1.4)^3^  2.4 (1.5)^8^  2.2 (1.5)^13^  2.1 (1.5)^18^  2.3 (1.5)^23^  2.6 (1.2)^28^  1.2 (0.9)^33^  1.3 (0.9)^38^  1.4 (0.9)^43^ | 1.8 (1.4)^4^  2.1 (1.5)^9^  2.0 (1.5)^14^  1.9 (1.5)^19^  2.1 (1.5)^24^  2.6 (1.2)^29^  1.0 (0.9)^34^  1.0 (0.9)^39^  1.1 (0.9)^44^ | 2.1 (1.5)^5^  2.5 (1.6)^10^  2.3 (1.5)^15^  2.1 (1.5)^20^  2.4 (1.4)^25^  2.5 (1.1)^30^  1.3 (0.9)^35^  1.4 (0.9)^40^  1.7 (1.0)^45^ |
| **Psychological**  Mean CESD-8 score  [0 to 8] (SD): Wave 1  Wave 2  Wave 4  Mean cognitive impairment score  [0 to 49] (SD): Wave 1  Wave 2  Wave 4 | 1.5 (1.9)^46^  1.7 (2.0)^51^  1.5 (1.9)^56^  25.0 (6.6)^61^  27.5 (6.3)^66^  25.6 (6.8)^71^ | 1.2 (1.7)^47^  1.3 (1.7)^52^  1.1 (1.7)^57^  25.2 (6.7)^62^  26.3 (6.4)^67^  25.8 (6.6)^72^ | 1.8 (2.0)^48^  1.9 (2.1)^53^  1.8 (2.0)^58^  24.7 (6.5)^63^  25.5 (6.5)^68^  25.5 (6.9)^73^ | 1.4 (1.9)^49^  1.5 (1.9)^54^  1.3 (1.8)^59^  23.1 (6.2)^64^  24.1 (6.0)^69^  24.0 (6.2)^74^ | 1.7 (2.0)^50^  1.9 (2.0)^55^  1.8 (2.0)^60^  27.5 (6.3)^65^  28.4 (6.3)^70^  28.6 (6.7)^75^ |
| **Social**  Mean poor social support score  [0 to 54] (SD): Wave 1  Wave 2  Wave 4  Mean low poor social integration score  [0 to 14] (SD): Wave 1  Wave 2  Wave 4 | 13.6 (7.1)^76^  13.7 (7.0)^81^  13.6 (6.9)^86^  6.6 (2.5)^91^  6.6 (2.5)^96^  6.8 (2.5)^101^ | 14.9 (7.1)^77^  14.7 (7.0)^82^  14.5 (7.1)^87^  6.7 (2.6)^92^  6.7 (2.6)^97^  6.8 (2.6)^102^ | 12.6 (6.8)^78^  12.9 (6.8)^83^  12.8 (6.6)^88^  6.5 (2.5)^93^  6.5 (2.5)^98^  6.7 (2.4)^103^ | 13.8 (7.1)^79^  13.9 (7.0)^84^  13.7 (7.0)^89^  6.3 (2.5)^94^  6.4 (2.5)^99^  6.6 (2.4)^104^ | 13.3 (7.0)^80^  13.3 (6.8)^85^  13.2 (6.6)^90^  6.9 (2.6)^95^  7.0 (2.6)^100^  7.2 (2.6)^105^ |

*Note*

CESD-8: Center for Epidemiologic Studies Depression Scale (8 items)

N = ^1^4,608 ^2^2,052 ^3^2,556 ^4^2,617 ^5^1,991^6^3,115 ^7^1,350 ^8^1,765 ^9^1,909 ^10^1,206 ^11^2,400 ^12^1,022 ^13^1,378 ^14^1,642 ^15^758 ^16^2,319 ^17^1,064 ^18^1,255 ^19^1,436 ^20^883 ^21^1,504 ^22^668 ^23^836 ^24^1,034 ^25^470 ^26^996 ^27^440 ^28^556 ^29^763 ^30^233 ^31^4,572 ^32^2,036 ^33^2,536 ^34^2,597 ^35^1,975 ^36^4,567 ^37^2,032 ^38^2,535 ^39^2,611 ^40^1,956 ^41^3,125 ^42^1,355 ^43^1,770 ^44^1,915 ^45^1,210 ^46^4,484 ^47^1,999 ^48^2,485 ^49^2,4557 ^50^1,927 ^51^4,479 ^52^1,987 ^53^2,492 ^54^2,586 ^55^1,893 ^56^2,960 ^57^1,285 ^58^1,675 ^59^1,859 ^60^1,101 ^61^4,371 ^62^1,954 ^63^2,417 ^64^2,503 ^65^1,868 ^66^4,349 ^67^1,946 ^68^2,403 ^69^2,546 ^70^1,803 ^71^2,605 ^72^1,145 ^73^1,460 ^74^1,680 ^75^925 ^76^3,530 ^77^1,605 ^78^1,925 ^79^2,105 ^80^1,425 ^81^3,339 ^82^1,529 ^83^1,810 ^84^2,068 ^85^1,271 ^86^2,236 ^87^1,000 ^88^1,236 ^89^1,473 ^90^763 ^91^3,597 ^92^1,641 ^93^1,956 ^94^2,144 ^95^1,453 ^96^3,267 ^97^1,506 ^98^1,761 ^99^2,035 ^100^1,232 ^101^2,184 ^102^984 ^103^1,200 ^104^1,445 ^105^739

**Table 8. Moderation of mediated effects on future physical frailty: standardized coefficients from latent growth curve models**

|  | **Low physical activity** | | **Depressive symptoms** | | **Poor social support** | | **Poor social integration** | |
| --- | --- | --- | --- | --- | --- | --- | --- | --- |
|  | **Below mean ^a^** | **Above mean ^b^** | **Below mean ^c^** | **Above mean ^d^** | **Below mean ^e^** | **Above mean ^f^** | **Below mean ^g^** | **Above mean ^h^** |
| Indirect effect on physical frailty through chronic disease: | | | | | | | | |
| Low physical activity | 0.027** | 0.053** | 0.051** | 0.052** | 0.054** | 0.052** | 0.051** | 0.054** |
| Depressive symptoms | 0.039** | 0.030** | 0.037** | 0.024** | 0.042** | 0.033** | 0.042** | 0.032** |
| Cognitive impairment | 0.013** | 0.012 | 0.016** | 0.014 | 0.020** | 0.012* | 0.023** | 0.014* |
| Poor social support | 0.007 | 0.010 | 0.006 | 0.007 | 0.005 | 0.006 | 0.004Ɨ | 0.016*Ɨ |
| Poor social integration | -0.004 | -0.007 | -0.002 | -0.011 | -0.004 | -0.004 | -0.003 | -0.007 |
| Indirect effect on physical frailty through allostatic load: | | | | | | | | |
| Low physical activity | 0.005* | 0.003 | 0.008* | 0.005 | 0.010* | 0.006* | 0.007 | 0.006* |
| Depressive symptoms | 0.002* | 0.001 | 0.003* | 0.002 | 0.003* | 0.002* | 0.001 | 0.003* |
| Cognitive impairment | 0.003 | 0.004 | 0.003* | 0.005 | 0.007* | 0.002 | 0.002 | 0.006* |
| Poor social support | 0.002 | -0.001 | 0.001 | 0.001 | 0.002 | <0.001 | 0.002 | 0.001* |
| Poor social integration | <0.001 | 0.001 | -0.001 | 0.004 | 0.000 | 0.001 | 0.001 | <0.001 |

*Note*

* Indicates p-value <0.05 but >=0.0125

** Indicates p-value <0.0125 (to take into account Bonferroni’s correction for 4 comparison models)

Ɨ Indicates moderation with p-value <0.05 but >=0.0125

N=4,638 (all), 2,070 (male), 2,568 (female), 2,643 (less than 75 years old), and 1,995 (at least 75 years old)

For subgroups, N = ^a^2,819 ^b^1,819 ^c^3,324 ^d^1,314 ^e^2,275 ^f^2,363 ^g^2,244 ^h^2,394
